# The Altered Metabolic Molecular Signatures Contribute to the RAD001 Resistance in Gastric Neuroendocrine Tumor

**DOI:** 10.3389/fonc.2020.00546

**Published:** 2020-04-21

**Authors:** Jie Pan, Qi Bao, Georg Enders

**Affiliations:** ^1^Department of Endocrinology and Metabolism, School of Medicine, Second Affiliated Hospital of Zhejiang University, Hangzhou, China; ^2^Institution of Gastroenterology, Zhejiang University, Hangzhou, China; ^3^Walter Brendel Centre of Experimental Medicine, University of Munich, Munich, Germany; ^4^Department of Plastic and Reconstructive Surgery, School of Medicine, Second Affiliated Hospital of Zhejiang University, Hangzhou, China

**Keywords:** mTOR inhibitor, neuroendocrine tumor, resistance, metabolism, PI(3)K–Akt–mTOR signaling, MEK/ERK signaling, senescence

## Abstract

Although the inhibition of mTOR is a promising treatment for neuroendocrine tumors, several questions are still open for cell specificity and resistance. With the newly characterized gastric neuroendocrine tumor mouse model (CEA424-SV40 T antigen transgenic mice), the anti-tumor efficiency of RAD001 (Everolimus) was tested both *in vitro* and *in vivo*. Tumor samples were analyzed for the expression of RNA by cDNA microarrays and also signaling pathways to get more details on the local surviving or selected cells. RAD001 treatment dramatically slowed down tumor growth and prolonged the animals' survival. This inhibitory effect has a preference for tumor cells since gastrointestinal hormone and neuroendocrine tumor specific markers were more reduced than the epithelial ones. While phosphorylation of p70S6K was almost completely blocked both *in vitro* and *in vivo*, the phosphorylation of 4EBP1 was only partially inhibited *in vitro* and unaffected *in vivo*. RAD001 treatment induced feedback activation of metabolism related pathways like PI(3)K–Akt–mTOR and MEK/ERK signalings. An induction of senescence as well as differential expression of genes responsible for metabolism was also observed, which highlighted the contribution of metabolic molecular signatures to the escape of the tumor cells from the treatment. Together, our data revealed efficient anti-tumor ability of RAD001 in a new gastric neuroendocrine tumor mouse model system and offered new insights into the clinical aspects of the incomplete elimination of tumor cells in patients treated.

## Introduction

Gastroenteropancreatic neuroendocrine tumors (GEP-NETs) comprise a highly heterogeneous group of tumors with strikingly various clinical behaviors (1). Although they are considered to be much rarer than adenocarcinomas (1) and represent only up to 2% of all malignancies, their incidence has been increasing exponentially over the past decades, and are reported as the second most common malignancy of the gastrointestinal tract, right after colorectal cancer (2–5). Regardless of the improved disease awareness and diagnostic techniques, the overall 5-years survival has not changed tremendously since 1973 (1). Thus, model systems for studying disease pathogenesis and screening novel treatment approach are urgently needed to improve clinical management.

**Figure 1:**
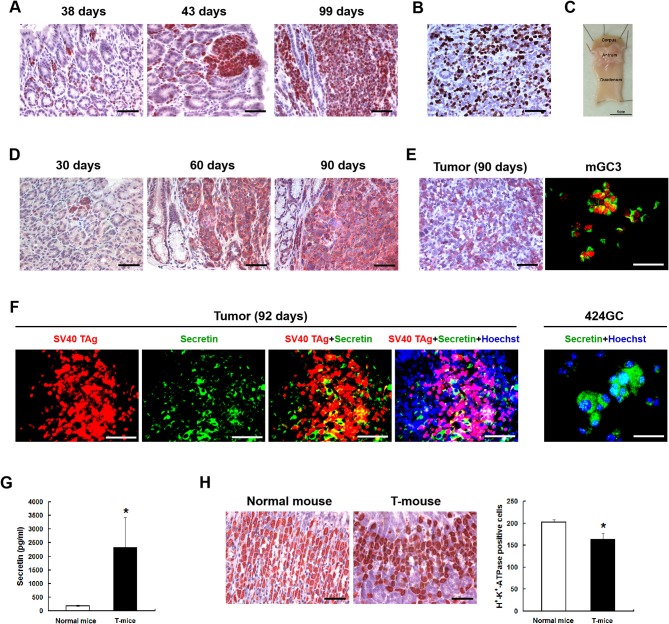
The neuroendocrine phenotype of the CEA424-SV40 T antigen transgenic mouse model. **(A)** Immunohistochemistry for SV40-TAg was applied to stomach sections from mice of different age to identify the tumor areas. **(B)** Ki67 staining in the tumor area of a 107 days old mouse. Macroscopic picture of the stomach from this mouse was shown in **(C,D)** Immunohistochemistry for chromogranin B on stomach sections from 30, 60, and 90 days old CEA424-SV40 TAg transgenic mice. **(E)** Left: immunohistochemistry for glucagon on stomach section from a 90 days old CEA424-SV40 TAg transgenic mouse. Right: SV40 TAg and glucagon double staining on cell line mGC3. SV40 TAg: red (Alexa-546); glucagon: green (Alexa-488). **(F)** Left: SV40 TAg and secretin double staining on stomach section from a 92 days old CEA424-SV40 TAg transgenic mouse. Right: immunofluorescent staining for secretin in cell line 424GC. SV40 TAg: red (Alexa-546); secretin: green (Alexa-488); nuclei were stained with Hoechst 33342. **(G)** ELISA analysis of secretin level in the plasma of 90-days-old CEA424-SV40 TAg mice and non-transgenic mice. T-mice: CEA424-SV40 TAg transgenic mice; *n* = 4 in each group; ^*^p < 0.05 vs. control. **(H)** As secretin functions as a feedback inhibitor of gastric acid secretion, elevated secretin hormone level leads to reduced acid producing cell numbers. Left: immunohistochemical staining for H^+^-K^+^-ATPase on stomachs of CEA424-SV40 TAg transgenic mice and normal mice. Right: statistical analysis for H^+^-K^+^-ATPase positive cell number. *n* = 5 in each group, ^*^p < 0.0005 vs. control. Scale bars in the staining pictures: 50 μm.

Good models should show a particular and stable phenotype which mimics the clinical situation as close as possible and can be easily reproduced. There are some genetically engineered murine models available for neuroendocrine tumors in pancrease (6–10), prostate (11–13), and colon (14), while there are few well-described model for NETs in stomach, like Apt4b-SV40 TAg transgenic mouse (15) and knockin mouse model for the ATP4a(R703C) mutation (16, 17). With a carcinoembryonic antigen (CEA) minimal promoter and the SV40 large T antigen the CEA424-SV40 T antigen transgenic mouse model was generated, which was originally designed to produce a model system for colorectal cancer. Interestingly enough, one subline of these mice developed high proliferative tumors in the antrum region of the stomach in 100% of the offspring (18), which we further characterized as neuroendocrine carcinoma (19). In addition, several cell lines were also established from the primary tumor (20) which we further characterized and found to have a similar cDNA profile like the tumor samples and express chromogranin A, B, as well as secretin and glucagon (19) (Figure 1). So they clearly resemble the tumor originating and located in the antrum of the stomach in CEA-T antigen heterozygous mice and offer good *in vitro* capabilities to screen drugs.

From several drugs tested, mTOR inhibitors showed a great efficacy in stopping tumor cell growth in our cell lines. The activation of the mTOR pathway is a hallmark of several different tumors, including GEP-NETs (21–24). Neuroendocrine tumors were among the first tumors to be treated with mTOR inhibition. More recent clinical studies have shown an impressive improvement on the median progression-free survival although complete remission was more the exception than the rule (25–28). The question therefore remains, whether only selected tumors are sensitive, or tumor cells are selected and/or develop resistance. There have been studies which indicate that loss of the p70S6K-mediated negative feedback loop on the PI(3)K–Akt–mTOR pathway might limit the antitumor effects induced by mTOR inhibitors (29). while more recent studies reported that negative or lower expression of mTOR, p70S6K, AKT, ERK1/2 were an indicator of RAD001 resistance (30). Thus, the exact resistant mechanism underlying is still unclear. In this study, the anti-tumor efficiency of mTOR inhibitor RAD001 (Everolimus) was tested *in vitro* and *in vivo* with special emphasis on signaling pathways to get more details on the local surviving or selected cells.

## Results

### RAD001 Effectively Inhibits Tumor Cell Growth Both *in vitro* and *in vivo*

**Figure 2:**
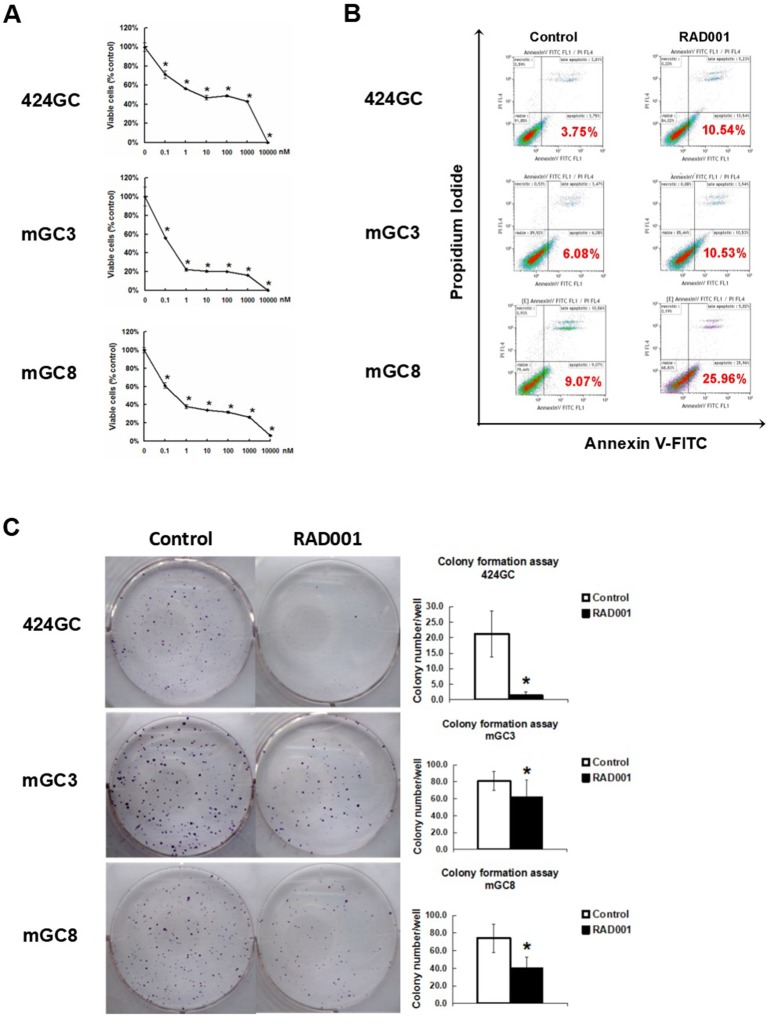
mTOR inhibitor RAD001 induced anti-tumor effects *in vitro*. **(A)** RAD001 efficiently inhibited cell proliferation in a dose dependent manner. WST-1 absorption was tested after 72 h treatment. The experiments were performed in triplicate. *p < 0.05 vs. control. **(B)** Cells treated with 100 nM RAD001 for 72 h were tested for apoptotic rate. Higher apoptotic rates were observed in the cell samples treated with RAD001. **(C)** Cells treated with 100 nM RAD001 for 72 h were seeded into 6-well plate for colony formation. Decreased clone numbers were observed in the treated group (*n* = 6 for each group, *p < 0.05: RAD001 treated group vs. control group).

The neuroendocrine phenotype of the CEA424-SV40 TAg transgenic mouse model system was described previously by our team (19). Genes specific for neuroendocrine family were significantly up-regulated in the tumors spontaneously developed in the antral region of the stomach as well as in cell line 424GC, which is derived from the primary tumor (19). In Figure 1, we could further show that neuroendocrine markers chromogranin B, glucagon and secretin were highly expressed in tumor cells and a significantly elevated hormone level of secretin was also detected in the plasma of tumor bearing mice comparing to non-transgenic littermates. According to the features described, especially the high Ki-67 index, which is over 50%, this mouse model most probably refers to gastric neuroendrocrine tumors type 3 (Figure 1). In tumors as well as in GC-cell lines, activation of the mTOR pathway could be constantly shown. Thus, mTOR inhibition seems to be an adequate therapeutic protocol for testing the role of mTOR in the growth neuroendocrine tumors. All cell lines tested showed a dose dependent inhibition of cell proliferation. It was interesting to note, that this inhibition followed a stepwise course with a plateau phase between 1 nM and 1 μM. A further increase of the drug concentration to 10 μM however stopped the proliferation completely (Figure 2A). While the WST-1 assay shows a more transient effect of the drugs, a colony formation assay can display the long term effects of the treatment on cell proliferation. Thus, cell lines treated with 100 nM RAD001 for 72 h were seeded into 6-well plate for colony formation. After 3 weeks cultivation, colonies formed were fixed by methanol and stained with crystal violet. For all the three drugs, 72 h treatment significantly inhibited the colony forming ability of all the three cell lines (Figure 2C). Seventy two hours treatment with RAD001 also induced higher apoptotic rate in all the three cell lines (Figure 2B).

**Figure 3:**
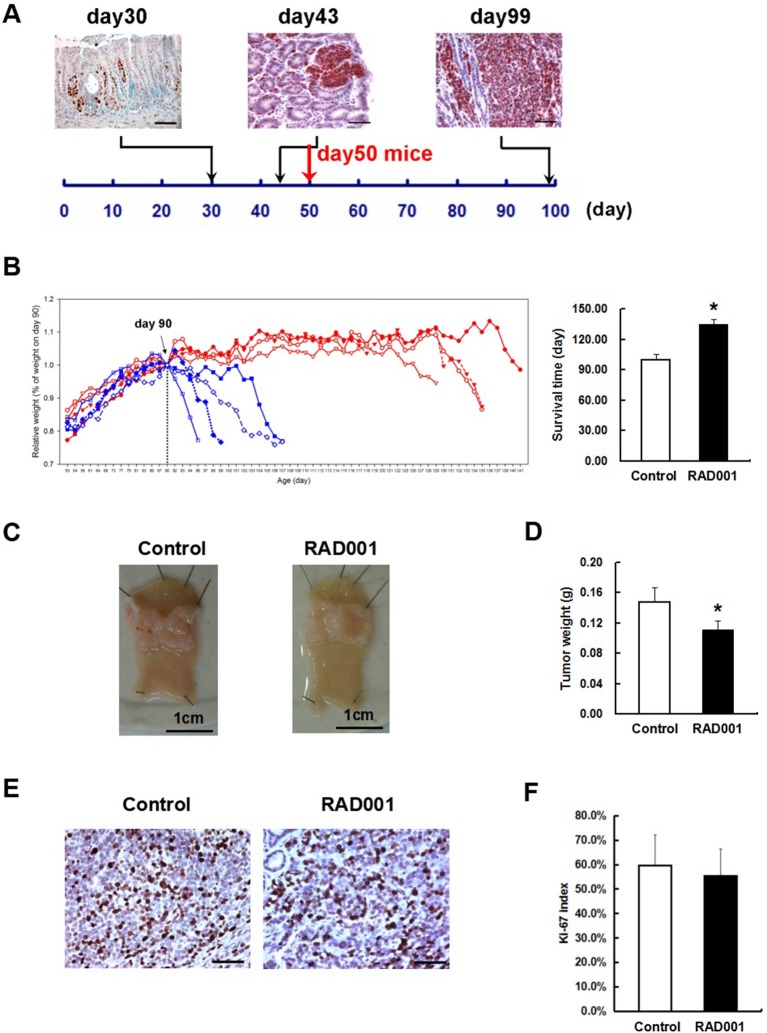
RAD001 treatment prolonged the survival time of the CEA424-SV40 TAg transgenic mice. **(A)** SV40-TAg staining was applied to stomach sections from mice of different age. *In vivo* experiment started with mice of day-50, when tumors were well-formed. Scale bars: 50 μm. **(B)** Left: weight curves of the CEA424-SV40 TAg transgenic mice. Blue curve: control group; red curve: RAD001 treated group. Right: mean age of the CEA424-SV40 TAg transgenic mice when they lost 20% of the peak weight (*p < 0.0001: RAD001 treated group vs. control group). **(C)** Macroscopic picture of the stomachs from control mice and RAD001 treated mice after 48 days of treatment with placebo or RAD001. Mean tumor weight of these two groups was shown in **(D)** (*p < 0.05: RAD001 treated group vs. control group). **(E)** An example of the Ki67 staining on stomach sections from control mice and RAD001 treated mice. Scale bars: 50 μm. No significant difference was found for the Ki-67 index in the two groups **(F)**.

From these inhibition experiments and reports from the literature we selected a concentration of 10 mg/kg/BW for treating animals. Beginning at day 50, when transgenic mice have distinct tumors in the antrum (Figure 3A), animals were treated with 10 mg/kg RAD001 or placebo by gavage once per day from day 1 to 5 every week. As a measure of effectiveness, the weight of the animals was monitored daily. In the first experiment, the difference of the survival time was compared between the control group and RAD001 treated mice. According to the animal right legal restrictions by the government, all the mice were sacrificed when they lost 20% of their peak weight or severe behavioral change was observed, which also clearly indicates that the tumor was large enough to obstruct the passage of the food. The feeding of RAD001 or placebo continued until the mice were sacrificed. The average starting weight in this experiment was 18.56 ± 3.22 g for control group and 17.22 ± 2.14 g for RAD001 treated group (*p* > 0.05, *n* = 4) (Figure S4). Mice in the control group started to lose weight at around day 90–100, while mice in the RAD001 treated group showed a comparable weight loss not before day 126–136. The mTOR inhibition could clearly slow down tumor growth and significantly extend the survival of animals by 35 days in average compared with sham treated controls (Figure 3B). In the second experimental setting in which all the mice were sacrificed on day 98 (that is the day when the first control mouse started to lose weight) we found that tumor weight was significantly reduced in the RAD001 treated group when samples were taken at the same age of animals (Figures 3C,D). This situation reflects quite precisely the clinical observation on growth retardation and validates that neuroendocrine tumors are responsive to mTOR inhibition. This also made it obvious, that the effect of the mTOR inhibition was a slow down of the proliferation in CEA424-SV40 T antigen transgenic mice, but not a cytotoxic effect on the tumor cells and a cure for the mice. The tumor cells may lose the sensitivity to the inhibitory effects induced by RAD001 on typical mTOR signaling pathways.

### The Discrepant Inhibitory Effect of RAD001 on mTOR Signaling

**Figure 4:**
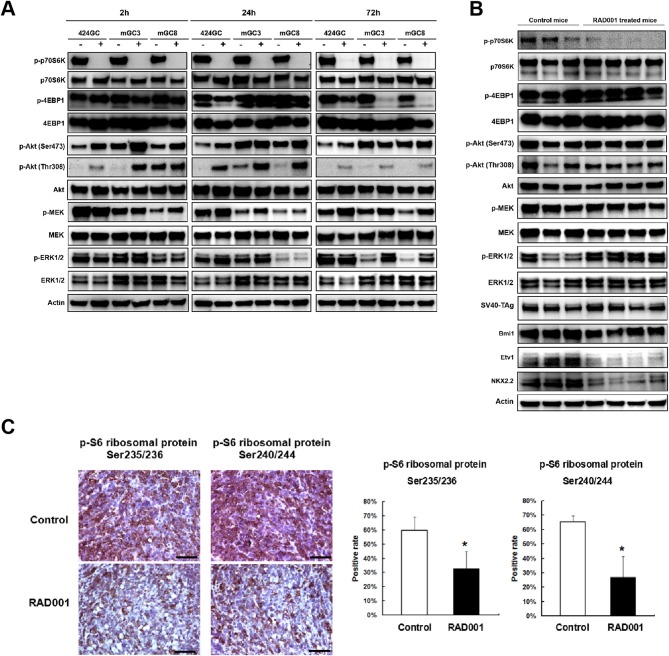
RAD001 efficiently inhibited the mTOR-p70S6K signaling while inducing a feedback activation of PI(3)K–Akt–mTOR and MEK/ERK pathways. **(A)** The expression of phosphorylated and total p70S6K, 4EBP1, Akt, MEK, and ERK1/2 was evaluated in the primary tumor cell lines and cells treated with RAD001 for 2, 24, and 72 h with western blot. The same analysis was applied to the tumor tissues from RAD001 treated mice and control mice after 48 days of treatment with RAD001 or placebo **(B)**. **(C)** Staining for p-S6 ribosomal protein was applied to tumor sections from RAD001 treated mice and control mice. Scale bars: 50 μm. Statistical analysis for p-S6 ribosomal protein positive cell rate is shown on the right. *p < 0.05: RAD001 treated group vs. control group.

Western blot and immunostaining showed that both the cell lines and the tumor tissue highly express the downstream target of mTOR (Figure 4), indicating that in the CEA424-SV40 TAg gastric tumor and the cell lines mTOR pathway is highly active. Treatment of cells for 2, 24, and 72 h with RAD001 completely suppressed the phosphorylated form of p70S6K (Figure 4A and Figure S1). This effect could also be seen in tissue samples from RAD001 treated animals reflecting the power of mTOR inhibition *in vivo* (Figure 4B and Figure S2). In addition, the expression of phospho-S6 ribosomal protein, which is a downstream target of p70S6K, was also reduced in the tumor tissue, showing further evidence of an effective mTOR inhibition, although this effect was not complete and could only lower the percentage of positive cells by about 50% (Figure 4C).

In addition, the phosphorylation of 4EBP1 was also studied as another mTORC1 substrate responsible for transcription and translation initiation of genes involved in cell growth. The effect of the mTOR inhibition could be followed quite easily in GC cell lines, although the time course was quite different from the p70S6K-inhibition. While treatment for 2 h had no effect on phospho-4EBP1 concentration in the cell lines, 424GC responded only weakly after 24 h while GC3 and GC8 offered the strongest inhibition after 72 h (Figure 4A). So, there is a time and cell line specific difference, which might be associated with a different responsiveness of the two latter lines. In accordance with this, the inhibitory effect on the phosphorylation of 4EBP1 could not be seen *in vivo* (Figure 4B). This may indicate that although both mTOR substrates can be suppressed by RAD001 *in vitro*, the anti-tumor effects observed in our CEA424-SV40 T antigen transgenic mouse model system may dependent more on the inhibition of p70S6K, or some other feedback mechanisms help rescue the tumor cells.

There is some controversy whether the preferential inhibition of the TORC1 pathway by Everolimus activates as a consequence the phosphorylation of AKT either at Ser473 by TORC2 or Thr308 by the PI3-pathway kinase PDPK1 and thus bypasses the inhibitory effects. In tissue samples, the phosphorylation of AKT at Ser473 was higher after mTOR inhibition (Figure 4B and Figure S2), while in the cell lines RAD001 induced Akt phosphorylation after 2 and 24 h stimulation at both sites (Figure 4A). This points to the importance of TORC2 and the PI3kinase pathway in the sequence of tumor development.

### Possible Escape Mechanisms Learned From the RAD001 Resistant Tumor Cells

**Figure 5:**
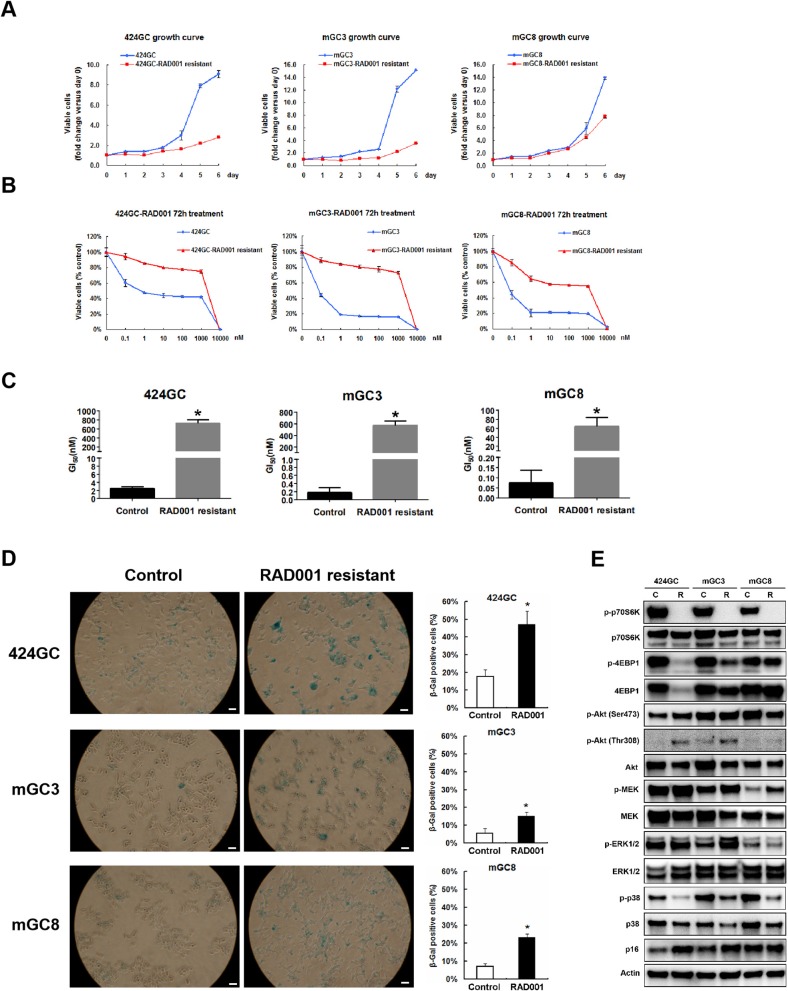
Study on the RAD001 resistant cell lines. **(A)** Growth curves of RAD001 resistant cell lines and the parental untreated cell lines. A slowed down proliferation was observed in the resistant cell lines comparing to the parental untreated cell lines even being cultured in RAD001 free medium. **(B)** Cell viability assay was applied to RAD001 resistant cell lines and the parental untreated cell lines. WST-1 absorption was tested after 72 h treatment. **(C)** The GI_50_s of the primary cell lines and RAD001 resistant cell lines (*p < 0.05: RAD001 resistant cell lines vs. primary cell lines, *n* = 3). Much higher GI_50_s were achieved in the resistant cell lines. **(D)** RAD001 resistant cell lines and their parental untreated cell lines were analyzed for acidic senescence-associated β-galactosidase activity. Higher percentage of β-gal positive cells was observed in the resistant cell liens. Scale bars: 100 μm. *p < 0.05: RAD001 resistant group vs. control group. **(E)** The expression of molecular markers was compared between the RAD001 resistant cell lines kept in 400nM RAD001 and the parental untreated cell lines by western blot.

The treatment in the model system made it obvious, that the effect of the mTOR inhibition was a slowdown of the proliferation in CEA424-SV40 T antigen transgenic mice, but not a cytotoxic effect on the tumor cells and a cure for the mice. And one fascinating result from the *in vivo* treatment is that, when the Ki67 index was evaluated at the time point of terminal tumor growth, no significant difference was found between controls and RAD001 treated animals (Figures 3E,F). So mTOR inhibition affected almost exclusively later steps in tumor cell survival. Thus, we started a selection of the cell lines *in vitro*. We selected GC cells *in vitro* by chronic RAD001 exposure at concentrations of up to 400 nM reflecting partially resistant cells. When tested acutely, these cells were less sensitive to RAD001 and even in the absence of RAD001 grew much slower than controls (Figures 5A,B). This effect could be shown in all three separate lines tested, although with slight differences. The calculations for GI_50_ in the resistant cell lines showed a 290-fold increase in 424GC (2.49 ± 0.42 nM for control cells and 722.51 ± 79.88 nM for resistant cells), a 3,168-fold increase in mGC3 (0.18 ± 0.12 nM for control cells and 573.46 ± 75.16 nM for resistant cells), and a 864-fold increase in mGC8 (0.07 ± 0.06 nM for control cells and 64.55 ± 19.11 nM for resistant cells) when compared to the primary cell lines (Figure 5C). The resistant cell lines could be stored in liquid nitrogen and brought back into culture after freezing and thawing cycles without significant change in the morphologic appearance. To check if the resistance aquired was stable, we thawed the resistant cell lines which had been stored in liquid nitrogen for more than 3 months and kept them in culture with medium without RAD001 for 4 weeks before the dose-reponse experiment for RAD001 was repeated. Persistent resistance was observed with GI_50_s of the same order of magnitude in all the three cell lines. When the typical response of these cells on the phosphorylation of mTOR target gene p70S6k was tested, it became clear that they were still responsive to RAD001 (Figure 5E and Figure S3). Interestingly enough, the 4EBP1 phosphorylation was not affected at all in mGC8 (Figure 5E). This was reminiscent of the response in tissue, that long term treatment of the animals with RAD001 blocked p70S6K phosphorylation but had only marginal effects on 4EBP1 phosphorylation (Figure 4B).

Besides, MEK/ERK signaling was also observed in RAD001 resistant cell lines. Up-regulated expression of phospho-MEK was observed in all the tested three RAD001 resistant cell lines while an induction of phospho-ERK1/2 was detected in mGC3-RAD001 resistant cell line (Figure 5E). Actually, the enhanced expression of phospho-ERK1/2 was also detected in the tumor tissues (Figure 4B and Figure S2) and cell line mGC3 and mGC8 after 72 h treatment with 100 nM RAD001 (Figure 4A). *In vitro* treatment of 2 and 24 h was not sufficient to induce a significant activation of this pathway. It is worth mention that the induction of p-Akt (Thr308) expression could still be observed in cell line 424GC and mGC3 after long term RAD001 treatment, although this effect was not as strong as seen in short term treatment of 2 and 24 h (Figure 5E).

**Figure 6:**
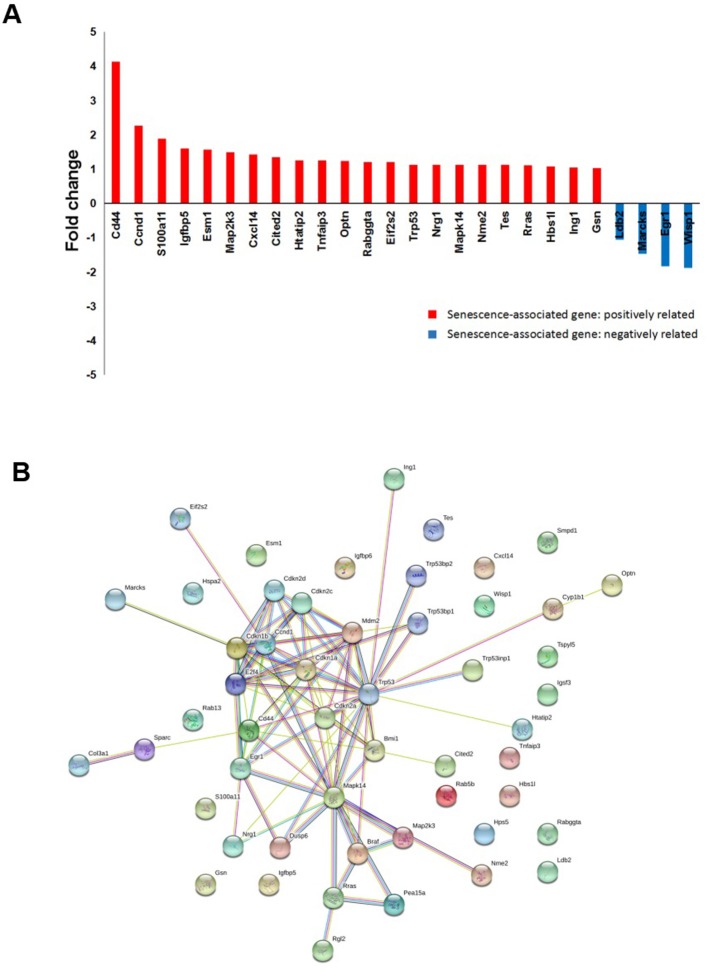
microarray analysis on tumor tissue samples for senescent signature. **(A)** cDNA-array analysis in the tumor tissues from RAD001 treated mice and control mice showed a slight induction of senescence associated genes (*p* < 0.05: RAD001 treated group vs. control group). **(B)** Senescent related genes differentially expressed between control group and RAD001 treated group (*p* < 0.05) were selected for STRING analysis. Protein-protein interaction was shown.

Another interesting finding from the RAD001 resistant cell lines is that they displayed a much stronger senescent phenotype compared with control cells as shown by β-galactosidase staining and p16 expression detection (Figures 5D,E). Indeed array analysis showed a slight induction of senescence associated genes (Figure 6A). Senescence related genes differentially expressed between the control mice and RAD001 treated mice were further analyzed by STRING database. Protein-protein interaction revealed a core function of the Cdkn gene family (Figure 6B). In addition a reduction of p38 phosphorylation indicates a state of dormancy (Figure 5E).

### Gene Expression Profiles of NETs During mTOR Inhibition

**Figure 7:**
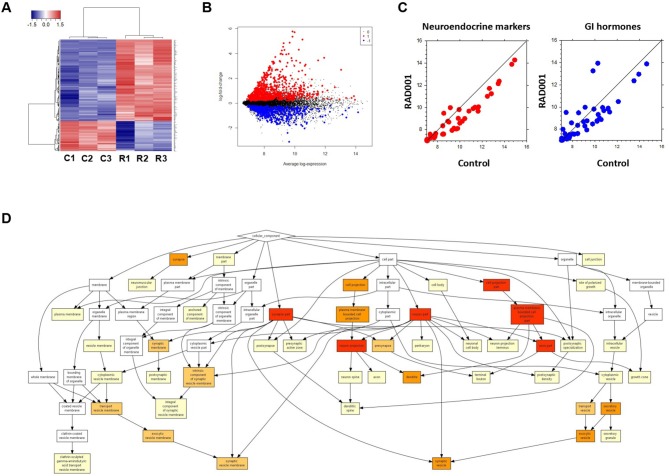
Microarray analysis on tumor tissue samples from mice treated with RAD001 or placebo. **(A)** Transcriptome analysis in the tumor tissue samples by cDNA-array. Three samples from each group were included. C, control mouse treated with placebo; R, mice treated with 10 mg/kg RAD001. Gene expression was analyzed by cluster 3.0 and TreeView. **(B)** From the whole array, 1,344 genes show differential expression between control group and RAD001 treated group (*p* < 0.05: RAD001 treated group vs. control group). Red: up-regulated for more than 2 folds; blue: down-regulated for more than 2 folds; black: fold change between −2 and 2. **(C)** A down regulation of genes specific for neuroendocrine tumors and gastrointestinal hormones was observed in mice treated with RAD001 comparing to control mice. Data are expressed as exponent only (log_2_). A position left from the diagonal indicates an increase, on the right an inhibition after RAD001 treatment. **(D)** GO analysis showed an enrichment of neuroendocrine related genes in the term of Cellular Component (CC).

Global analysis of gene expression offers the chance to get a broad overview on changes in processes induced by mTOR inhibition. Thus, we analyzed tumors from controls and RAD001 treated animals by cDNA-array (Supplementary Table 1). GO analysis of the whole gene-set revealed an enrichment of neuroendocrine related genes, which further confirmed the neuroendocrine phenotype of the tumors developed in our mice (Figure 7D). Based on the array data, 486 genes were found to be differentially expressed between the control group and RAD001 treated group (*p* < 0.05, fold change>2), in which 254 genes were up-regulated and 232 genes were down-regulated in RAD001 treated animals (Figures 7A,B and Supplementary Table 2). The increase most probably reflected a change in caspase 1, immune markers like CD44 and Ccl25 but also markers for an unspecific immune response like defensins, Lgals, Lyz1, and also mucin. Since mTOR inhibition initially has been used due to its immunosuppressive effects, this makes perfectly sense. A more intense analysis of the transcriptome in the tissue samples demonstrates, that mTOR inhibition selectively affects tumor cells since tumor specific target genes e.g., Sct, Chga, and Chgb or Thp1 were reduced (Figure 7C). Target genes were selected on the basis of their highest increase in developing tumors over time from controls to 90 days of age and shown by a recent paper (15). The expression of genes for gastrointestinal hormones were also found to be down regulated (Figure 7C). In contrast, expression of gastric epithelial cell markers e.g., mucins (Muc5ac), cadherin/cytokeratins remain unchanged or even go up in treated animals. While the expression of the SV40 large T antigen was only slightly down-regulated, Nkx2.2, a transcription factor highly expressed in tumor cells as well as in the parent tumor was considerably reduced in RAD001 treated animals. A similar reduction could be detected for Bmi1 and Etv1, the latter as one of the possible driving forces in tumor development in this particular tumor model (Figure 4B). All this points to the fact, that tumor cells are more susceptible to mTOR inhibition.

**Figure 8:**
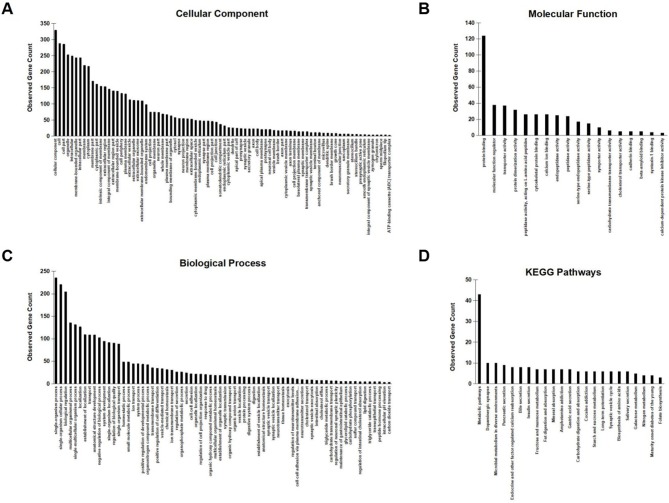
GO enrichment analysis and KEGG signaling pathway analysis. **(A–C)** Observed gene counts for each GO term were shown. **(D)** KEGG analysis identified 23 molecular pathways and gene counts for each mathway were shown.

**Figure 9:**
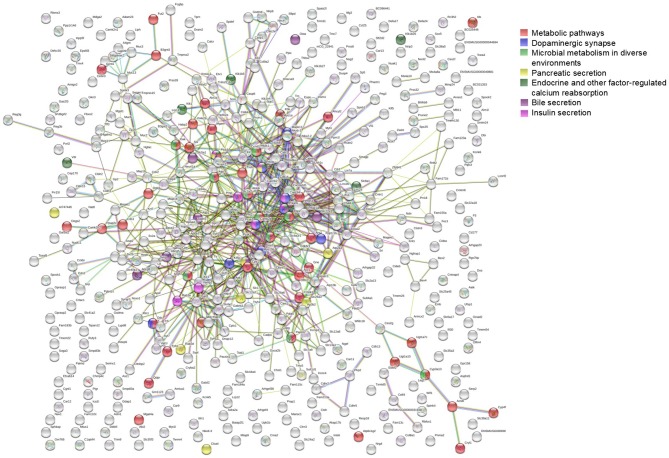
STRING analysis of the array data. Genes differentially expressed between control group and RAD001 treated group (*p* < 0.05, fold change>2) were selected for STRING analysis. Protein-protein interaction was shown. The top 7 pathways which contained the highest gene counts identified by KEGG analysis were labeled on the PPI map.

To further investigate the network characteristics of the genes differentially expressed after long-term treatment by RAD001, genes significantly up or down regulated by more than 2-folds were analyzed by STRING databese. The protein-protein interaction (PPI) network was showed in (Figure 9) (PPI enrichment *p*<1.0e−16). Functional enrichment analysis revealed 70 Biological Process (BP) GO terms, 82 Cellular Component (CC) GO terms, and 18 Molecular Function (MF) GO terms. The gene counts for each GO term were shown in (Figures 8A–C). The top MF GO term with highest gene counts was protein binding while the top BP GO term was single-organism process. KEGG significant enrichment showed that the top 7 pathway which included the largest number of genes was metabolic pathways, dopaminergic synapse, microbial metabolism in diverse environments, pancreatic secretion, endocrine, and other factor-regulated calcium reabsorption, bile secretion and insulin secretion, which highlighted the metabolic and neuroendocrine related pathways. These important pathways were also labeled in the PPI networks.

## Discussion

mTOR is a serine/threonine kinase belonging to the PI3K-related protein kinase (PIKK) family (31). As a key regulator for translational control, mTOR integrates various environmental cues to regulate host homeostasis (32). A growing body of evidence shows the link between activated mTOR signaling and tumor formation, including neuroendocrine tumors (33, 34). Mutations in genes of mTOR pathway were reported in 14% of pancreatic neuroendocrine tumors (p-NETs) (24). In a global gene expression microarray analysis for a large panel of pancreatic endocrine tumors, the tuberous sclerosis 2 (TSC2), and phosphatase and tensin homolog (PTEN), which are two well-defined mTOR inhibitors, were found to be downregulated in most of the primary tumors, and the disfunction of these two mTOR up-stream regulators could be recognized as a sigh for significantly shortened disease-free and overall survival (35). These observations were further confirmed by the phase III studies for p-NET with a significantly increased progress free survival in Everolimus treated patients (25–28), which leads to the approval of this drug by the Food and Drug Administration (FDA) and European Medicines Agency (EMA). Although an impressive improvement on the median progression-free survival has been shown by these studies, the efficancy of Everolimus is not strong enough to achieve a complete remission but only a delay of the disease progression. The underlying mechanisms of how the tumor cells manage to survive and escape from the treatment is not fully characterized. Our CEA424-SV40 T antigen gastric tumors and derived tumor cell lines highly express phosphorylated mTOR downstream substrates S6K and 4EBP1, indicating high mTOR activity. Thus, it provides a rationale for testing mTOR targeted therapy in the CEA424-SV40 TAg mouse model system both *in vitro* and *in vivo*, and this parallel strategy based on genetically engineered techniques is a big advance than single analysis on cell line or xenograft model system for key insights into the complexity of human disease.

In the current study, we are able to show that the activation of p70S6K, which is one major downstream target of the PI(3)K-Akt-mTOR pathway and responsible for cell proliferation, is significantly suppressed by the RAD001 treatment both *in vitro* and *in vivo*. In the cell lines, a complete elimination of phospho-p70S6K could already be achieved even after 2 h treatment, which clearly demonstrates the high efficiency of RAD001 treatment, although a potent effect on the 4EBP1 and MEK-ERK1/2 signaling needs a longer time of 72 h' treatment. Although RAD001 could efficiently inhibit cell proliferation *in vitro*, the anti-tumor effect induced by RAD001 *in vivo* was not strong enough to eliminate all the tumor cells. In the *in vivo* experiment, we chose a dose of 10 mg/kg for RAD001 treatment according to previous studies for RAD001 treatment on mice. The drug dose for human in mg/kg is usually multiplied by 12.3 to calculate mouse doses in mg/kg (36). The maximun Everolimus dose for human treatment in clinic is 10 mg (about 0.17 mg/kg for a patient of 60 kg), which is corresponding to 2 mg/kg for mouse. Although the dose in this study is higher compared to human studies, the mice tolerated this quite well. And even with such high drug dose, mice treated with RAD001 for almost 3 months still developed highly proliferative tumors which eventually led to the death of these mice. This indicated that these tumor cells *in vivo* became resistant to the treatment and reformed themselves to survive in the tough environment induced by the high concentration of RAD001. This is consistent with the situation in the clinic trails. Patients treated with Everolimus are not cured despite a prolonged progression-free survival. That is why we then established RAD001 resistant cell lines *in vitro* to investigate the altered molecular signals in the alive tumor cells after long-term RAD001 treatment. In previous study, Aristizabal Prada et al. also established independent Everolimus-resistant tumor cell lines with panNET cell line BON1. They chose a dose of 10 nM for selecting because the clinically relevant dose should be around 8–59 nM according to the plasma levels of everolimus in patients treated by 10 mg Everolimus daily (37). The dose we used to keep the resistant cell lines was 400 nM, which was also higher than the indicated clinically relevant dose. However, the selected tumor cells could be well-kept in culture with the high drug concentration and could survive after freezing and thawing cycles. The GI_50_s of these resistant cell lines were significantly elevated compared to the control cell lines and were still of the same order of magnitude after a drug holiday of 4 weeks, although the holiday was not long enough for a claim of a stable resistance as some other studies reported a complete reversal of resistance after a drug holiday of 12 weeks (38). It would be interesting to prolong the drug holiday for further confirmation of a persistant stable resistance as well as to repeat the *in vitro* and *in vivo* experiment in our model system with lower drug dose for better mimicking the clinical situation. Based on our current study, we point out that the incomplete inhibition of 4EBP1 phosphorylation may contribute to the limited therapeutic benefit from RAD001. Here, the mTORC1 substrate 4EBP1 seems to be not essential for the slowed down tumor development as the p-4EBP1 level remains intact after long term treatment *in vivo*, although in the fast growing cell lines (mGC3 and mGC8) a down regulation is observed after 72 h treatment. It is also interesting to note that when we prolong the treatment for 1 month, p-4EBP1 expression in mGC8 is restored. It indicated that long term RAD001 selection helped the mGC8 regain 4EBP1 phosphorylation. This cell-type-specific differential effects on p70S6K and 4EBP1 have also been shown by Choo et al. although in a totally different setup where mTOR activity was induced by serum or insulin after starvation instead of consistant oncogene driven mTOR activation as in our model system (39). Their study shows that S6K activity is potently inhibited by rapamycin throughout the duration of treatment, while 4EBP1 recovers in phosphorylation despite initial inhibition and they consider this reemerged 4EBP1 phosphorylation as one of the explanations for rapamycin resistance. In the case for our model system, the heterogeneity of the tumor cells may result in different response to the RAD001 treatment. As a consequence, cap-dependent translation could be differentially controlled in the presence of RAD001. Considering the crucial role of 4EBP1 in the regulation of tumor cell proliferation, its reinitiated phosphorylation could be a reasonable explanation for the maintained cap-dependent translation despite of mTORC1 inhibition.

To get more of an idea on a possible selection of resistant cells by mTOR inhibition, we did global analysis of gene expression and investigated the network characteristics of the genes differentially expressed after long-term treatment by RAD001. Here we noticed significantly altered metabolic molecular signatures. KEGG significant enrichment showed that “metabolic pathways” and “microbial metabolism in diverse environments” were among the top 3 pathways which included the largest number of genes. This makes sense as mTOR is a key regulator of multiple cellular mechanisms including metabolism. Cancer cells are well-documented to modifiy their metabolism networks to support survival and proliferation (40). RAD001 inhibited the mTORC1 downstream target p70S6K and thus achieved the anti-tumor effects both *in vitro* and *in vivo*. However, the activation of Akt by single RAD001 treatment due to the loss of p70S6K-mediated negative feedback loop on the PI(3)K–Akt–mTOR pathway helped the tumor cells to survive in the harsh conditions and finally led to the death of the tumor bearing mice. By phosphorylating IRS-1, S6K1 affects IRS-1 stability and thus blocks its effect on the PI(3)K–Akt signaling (25). In our model system, although no significant change was observed *in vivo*, an increase of phospho-Akt expression at Thr 308 was induced by RAD001 in the cell lines, which can be observed after 2, 24, and 72 h incubation. The RAD001 resistant cell lines also display higher level of phospho-Akt which means that this effect is consistent after long term selection *in vitro*, although the intensity of the induction tends to wane with time. On the other hand, we also checked the phosphorylation of AKT at another site Ser 473, which is usually induced by the activation of mTORC2. We found that in the cell lines the upregulation of phospho-Akt at this site is not so obvious and disappeared after 24 h treatment. So we think that the loss of S6K-mediated negative feedback on PI(3)K–Akt pathway contributes to the escape mechanism and a combination of PI(3)K or Akt inhibitor may induce even more potent anti-tumor effects. In fact, several studies have shown that PI3K inhibitors could re-establish everolimus sensitivity in human pancreatic neuroendocrine tumor cell lines (37, 38, 41). We have also tested PI3K inhibitor BEZ235 in the cell lines in preliminary experiment and observed potent anti-proliferative effect (data not shown). Further studies are needed to investigate if the combination of RAD001 and PI3K inhibitor helps overcome RAD001 resistance and enhance the therapeutic effect in gastric tumors with neuroendocrine phynotype and whether the treatment is well-tolerated.

Besides, we also observed a time course dependent activation of MEK/ERK signaling, which is another classic metabolism related pathway (42, 43), by RAD001 treatment. MEK/ERK signaling targets hundreds of proteins and has profound effects on growth of normal and malignant cells (44). The PI(3)K–Akt–mTOR and MEK/ERK pathway have also been reported as part of the genetic regulation of metabolism of other cancer types like lung cancer (45). There is also a cross regulation between these two pathways which builds up a complicated network critical for many aspects of tumorigenesis, drug resistance, and cellular senenscence (44, 46–48). These observations provide rational for a dual inhibitory strategy targeting both two signalings. Some preclinical studies have already shown that a combination of mTOR inhibitors with inhibitors targeting MEK or Raf leds to an enhanced response (49–52).

It is also worth mention that an induction of senenscence is observed in the RAD001 resistant cell lines. In theory, induction of senescence is considered as an anti-tumor mechanism and offers attractive therapeutic approach. It should be pointed out that this theory hinges on the principle that senescence is irreversible and the senescent cells are eventually eliminated by the host's defensive system (53, 54). However, there have been evidences of arrested malignant cells still being metabolically active and retaining the potential to recapture the proliferative capacity (54–56). Some studies also report that senescence can drive both degenerative and hyperplastic pathologiesstate and is associated with a poor therapeutic index in several tumor types (54, 57), which indicates that resistance may be induced by an accumulation of senescent cells and this senescent state may also serve as a protective mechanism for the tumor cells under stress. In our cultured cell lines, long term RAD001 treatment slowed down the proliferation with a higher rate of senescent cell. This arrested or relatively unresponsive state may help the tumor cells survive in the high drug concentration. On the other hand, it has also been reported that activated PI3K/AKT pathway can rapidly induce senescence in human fibroblasts and mTORC1 is an important mediator for this process (58). As mentioned above, we observed a slightly higher p-Akt expression in the RAD001 resistant cells which may also help to explain the induced senescence. Furthermore, our array analysis also points to significant alteration of pathways on metabolism, which supports the hypothesis that metabolically active cells are more sensitive to mTOR inhibition and tumor cells can adapt to this and respond by a slower proliferation and obtaining the senescent phenotype.

## Materials and Methods

### Cell Lines, Animals, and Drug

Three cell lines, 424GC, mGC3, mGC8, originally established by Robert Kammerer, were kindly provided by Prof. Wolfgang Zimmermann. These cell lines were derived from spontaneously developing tumors of three different, 13 weeks old CEA424 SV40 TAg transgenic mice (C57BL/6-Tg(CEACAM5-TAg)L5496Wzm), which have been described previously (20). Cells were maintained in RPMI1640 with fetal bovine serum gold (10% in final concentration), sodium pyruvate, non-essential amino acids, glutamine and β-mercaptoethanol. For storage, cell lines were frozen in liquid nitrogen with the freezing medium (10% DMSO medium, 35% fetal bovine serum gold, 55% culture medium). The CEA424-SV40 T antigen transgenic mice were kept and bred at the animal facility of the Walter Brendel Centre of Experimental Medicine. Animals were housed under conventional conditions with free access to food and water. Animal studies within this work were registered with and accredited by the local regulatory agency (Regierung von Oberbayern, 55.2-1-54-2532-47-11, Munich, Germany). RAD001 was provided by Novartis Pharma (Basel, Switzerland).

### Cell Viability Assay

Cells were seeded into 96-well plate at appropriate concentrations (424GC: 2 × 10^4^/well, mGC3: 1.5 × 10^4^/well, mGC8: 1.5 × 10^4^/well) in 100 μl culture medium described above and incubated overnight at 37°C_._ On the 2nd day, the previous medium was not discharged in order to avoid the loss of cells during the procedure of medium change. Instead, 100 μl fresh medium with drugs suspended at twice the test concentrations were added into each well. The cells were then incubated at 37°C for 72 h. At the end of the experiment, supernatant was removed and 100 μl fresh medium was added into each well. For cell proliferation assay, 10 μl WST-1 reagent (Roche, Germany) was added into each well. The plate was incubated at 37°C with 5% CO_2_ for 4 h. The absorbance of the samples against a background control as blank was measured with a microplate ELISA reader (Tecan, Germany). The wave length for measuring the absorbance of the formazan product was 460 nm. The reference wavelength was 690 nm.

### Colony Formation Assay

Cells treated with 100 nM RAD001 for 72 h were used for colony formation assay. The concentration of 100 nM RAD001 was chosen according to the literatures (46). Cells without any treatment were used as control. Cells of appropriate number were seeded into 6 well plate (424GC:1,000/well, mGC3: 800/well, mGC8: 800/well). Cultivation was lasted for 3 weeks and medium was changed every 4 days during this time. The clones were then fixed with methanol for 30 min and stained with 0.1% crystal violet in distilled water for 30 min. Subsequently, the clones were washed with tap water twice before the plates were photographed. The clone number was counted with Image J program.

### Flow Cytometry Analysis for Cell Apoptosis

Cells incubated with 100 nM RAD001 for 72 h were analyzed by FACS for cell apoptosis with Annexin V-FITC kit (Miltenyi Biotec, Germany) according to the manufacturer's instructions. Cells incubated with normal culture medium for the same period of time were used as control. The test was repeated twice to confirm the result.

### Senescence β-Galactosidase Cell Staining

β-Galactosidase staining was applied to 424GC, mGC3, mGC8, and their corresponding RAD001 resistant cell lines to detect senescent cells with a Senescence β-Galactosidase Staining Kit (Cell signaling, Germany). Briefly, cells were seeded into 24-well plate in appropriate number and cultured in culture medium without drug for 48 h. Prior to staining, cells were fixed with fixative solution provided by the kit for 10 min at room temperature. After washed with PBS, cells were incubated with β-Galactosidase staining solution at 37°C overnight in a dry incubator. The next day, β-Galactosidase Staining Solution was removed and the cells were overlayed with 70% glycerol.

### Animal Experiment

50 days old CEA424-SV40 T antigen transgenic mice were randomly assigned to treated group or control group. RAD001 agent together with placebo were provided by Novartis Pharma, Switzerland. The drug was supplied as a 2% microemulsion in certican. The placebo was also provided in the same solution. RAD001 stock solution and placebo was aliquoted and stored at −20°C in dark. Before feeding, one aliquot of RAD001 stock solution was thawed at room temperature and then diluted with distilled water to a final concentration of 1 mg/ml. After careful mixing on a vortex, the drug was given to mice by gavage within 1 h according to the datasheet provided by Novartis. The preparation of the placebo was the same as the RAD001. For feeding, 10 mg/kg (10 ml/kg) RAD001 or placebo was given by gavage daily from day 1 to 5 every week. The dose chosen was based on previous studies which also applied daily oral administration of the drug (59–61). In the first experiment, we tested the survival time when all tumors developed a terminal size. So all the mice were sacrificed when they lost 20% of the peak weight or severe behavioral change was observed. This was also the end point according to the legal restriction by animal right. To further analyze the effects of the mTOR inhibitor RAD001, a different set up was chosen. Instead of testing the survival time when all tumors developed a terminal size, a fixed time protocol was chosen. The experiment was stopped when the first control mouse started to lose weight. In another word, instead of being sacrificed when 20% of the peak weight was lost, all the mice in this experiment were ended at the same age (on day 98). Four 50 days old CEA424-SV40 TAg transgenic mice were included in the RAD001 treated group and three of the same age in the placebo group. 10 mg/kg RAD001 or placebo was given orally once per day from day 1 to 5 every week as before. Weight was monitored daily, as in the first set up. Tumor tissue harvested for western blot were all from the second experiment.

### Immunohistochemistry

The stomachs taken from mice were opened in the minor curvature, fixed in 4% formaldehyde for 2 h at room temperature, dehydrated and embedded in paraffin wax. 2–3 μm sections were deparaffinized in xylene, and rehydrated in a graded series of ethanol. Antigen retrieval was performed when necessary in boiling natrium citrate buffer (PH6.0) for 15 min. Antibodies directed at Glucagon (Cell signaling,1:100), Chromogranin B (Santa Cruz, 1:100), SV40 T Ag (Santa Cruz, 1:500), Ki-67 (Dako, 1:50), Hydrogen Potassium ATPase Beta (Abcam, 1:1000), Phospho-S6 Ribosomal Protein (Ser235/236) (Cell signaling, 1:100), Phospho-S6 Ribosomal Protein (Ser240/244) (Cell signaling, 1:800), were applied to the sections for 2 h incubation at room temperature. Sections were then treated with HRP-coupled secondary antibodies (ImmPRESS Anti-Rabbit Ig Polymer Detection Kit, Vector Labs) or biotin-conjugated secondary antibody followed by avidin-biotin-peroxidase complex (VECTASTAIN Elite ABC Kit, Vector Labs). Primary antibodies were visualized by AEC or DAB as substrate.

### Immunofluorescence

Tissue sections from the mice stomach were prepared as above. Antibodies directed at SV40 T Ag (Santa Cruz, 1:500), Glucagon (Cell signaling,1:100), Secretin (Biozol, 1:100) were used for primary incubation (2 h, room temperature). Sections were then reacted with appropriate secondary antibodies for 1 h at room temperature and counterstained with Hoechst33342.

### Protein Extraction and Western Blotting

Protein extraction was performed with PhosphoSafe™ Extraction Reagent (Novagen, Germany) according to the manufacturer's instructions. Proteins from cell and tissue lysates were resolved by polyacrylamide gel electrophoresis, transferred to nitrocellulose membrane in a semidry transfer unit, and blocked in 5% non-fat milk. Primary antibodies used were Actin (1:1000, Santa Cruz, USA), NKX2-2 (1:16000, Aviva Systems Biology, USA), Bmi1 (1:1000 Abcam, UK), Etv1 (1:1500, Abcam, UK), SV40 T Ag (v-300) (1:800, sc-20800) (Santa Cruz, USA), p16 (1:1000, Santa Cruz, USA), Phospho-p38 (1:1000, Thr180/Tyr182) (Cell signaling, Germany), p38 (1:1000, Cell signaling, Germany), Phospho-p70S6 Kinase (Thr389) (1:1000, Cell signaling, Germany), p70S6 Kinase (1:1000, Cell signaling, Germany), Phospho-4EBP1 (1:1000, Cell signaling, Germany), 4EBP1 (1:1000, Cell signaling, Germany), phospho-Akt (Thr308) (1:1000, Cell signaling, Germany), phospho-Akt (Ser473) (1:1000, Cell signaling, Germany), phospho-MEK (Ser217/Ser221) (1:1000, Cell signaling, Germany), MEK (1:1000, Cell signaling, Germany), phospho-ERK1/2 (Thr202/Tyr204) (1:1000, Cell signaling, Germany), ERK (1:1000, Cell signaling, Germany). The primary antibodies were diluted with 5% bovine serum albumin in PBS with tween. The membrane was incubated with primary antibodies overnight at 4°C on a rotating platform, washed and incubated in the corresponding horseradish peroxidase (HRP)-conjugated secondary antibodies for 1 h at room temperature. The detection of the HRP coupled antibodies was carried out with SuperSignal® West Femto Maximum Sensitivity Substrate (Thermo Scientific, USA). The signal was detected with a Hamamatsu Aequoria system.

### RNA Isolation and Microarray Analysis

Tumor tissue from the mice included in the first RAD001 experiment (as described above) was used for microarray analysis. Three tumor samples from each group were included. Total RNA isolation was performed with NucleoSpin RNA II kit (Macherey-Nagel, Düren, Germany) according to the manufacturer's instructions. Microarray analysis was performed as described previously (19). Genes differentially expressed between control and RAD001 treated group (*p* < 0.05, fold change >2) were further analyzed with STRING database (Version 10.5, https://string-db.org) for protein-protein interaction (PPI). Gene ontology (GO) terms and Kyoto Encyclopedia of Genes and Genomes (KEGG) pathways analysis were used to analyze the possible pathological molecular mechanism involved during the long term treatment of RAD001.

### Statistical Analysis

Statistical significance was assessed by comparing median values using the non-parametric Mann-Whitney-*U*-test for independent samples and *t*-test for random samples (Sigma plot 10.0). *p*<0.05 were considered significant.

## Conclusion

Taking together, the present work based on the CEA424-SV40 T antigen tumor mouse model system provides supportive evidence for the anti-tumor potential of the mTOR inhibitors. Single treatment of RAD001 efficiently inhibits cell proliferation *in vitro* and displays local effectiveness *in vivo*. Tumor progression is slowed down and the survival time of the tumor bearing mice is significantly prolonged. The “escape” mechanism of the tumor cells is currently not fully understood although the incomplete inhibition of 4EBP1 phosphorylation and the feedback activation of PI(3)K–Akt and MEK/ERK signaling as well as an induction of senescence may be possible explanations. Combination therapies may give new light on more successful clinical management. Thus, this new model system could be of great value not only for studies on the mechanisms of how SV40 TAg induces neuroendocrine tumors but also for exploring novel targeted therapy in a preclinical setting.

## Data Availability Statement

All datasets generated for this study are included in the article/Supplementary Material.

## Ethics Statement

The animal study was reviewed and approved by Regierung von Oberbayern, 55.2-1-54-2532-47-11, Munich, Germany.

## Author Contributions

GE and JP defined the research question and designed the whole study and wrote the manuscript together. JP did all the experiment under the guidance of GE. JP and QB did the data analysis. All authors read and approved the final manuscript.

## Conflict of Interest

The authors declare that the research was conducted in the absence of any commercial or financial relationships that could be construed as a potential conflict of interest. The reviewer SN declared a shared affiliation, with no collaboration, with one of the authors, GE, to the handling editor at the time of review.

## Supplementary Material

Gene expression profiles from the microarray analysis and the histograms of the western blot analyses were uploaded as Supplementary Material.

Figure S1 Histograms of the western blot analyses shown in Firure 4A for short term treatment of 2h, 24h and 72h in the cell line 424GC, mGC3 and mGC8. Blue: control group; red: cells treated by 100nM RAD001.

Figure S2 Histograms of the western blot analyses shown in Figure 4B for RAD001 treatment in vivo. The expression of phosphorylated and total p70S6K, 4EBP1, Akt, MEK and ERK1/2, as well as the expression of SV40 TAg, Bmi1, Etvi and Nkx2.2 were evaluated in the tumor tissues from RAD001 treated mice and control mice after 48 days of treatment with RAD001 or placebo (^*^*p*<0.05: RAD001 treated group versus control group).

Figure S3 Histograms of the western blot analyses between primary cell lines and RAD001 resistant cell lines. The expression of molecular markers was compared between the RAD001 resistant cell lines kept in 400nM RAD001 and the parental untreated cell lines.

Figure S4 The mice weight at the beginning of the treatment. There was no significant difference of the mice weight between the control group and RAD001 treated group in both the first experiment and the second experiment.
