# Supplementary figures and images for: The Altered Metabolic Molecular Signatures Contribute to the RAD001 Resistance in Gastric Neuroendocrine Tumor
Source: Front Oncol. 2020 Apr 21;10:546. doi: 10.3389/fonc.2020.00546 (PMC7186336; doi:10.3389/fonc.2020.00546)

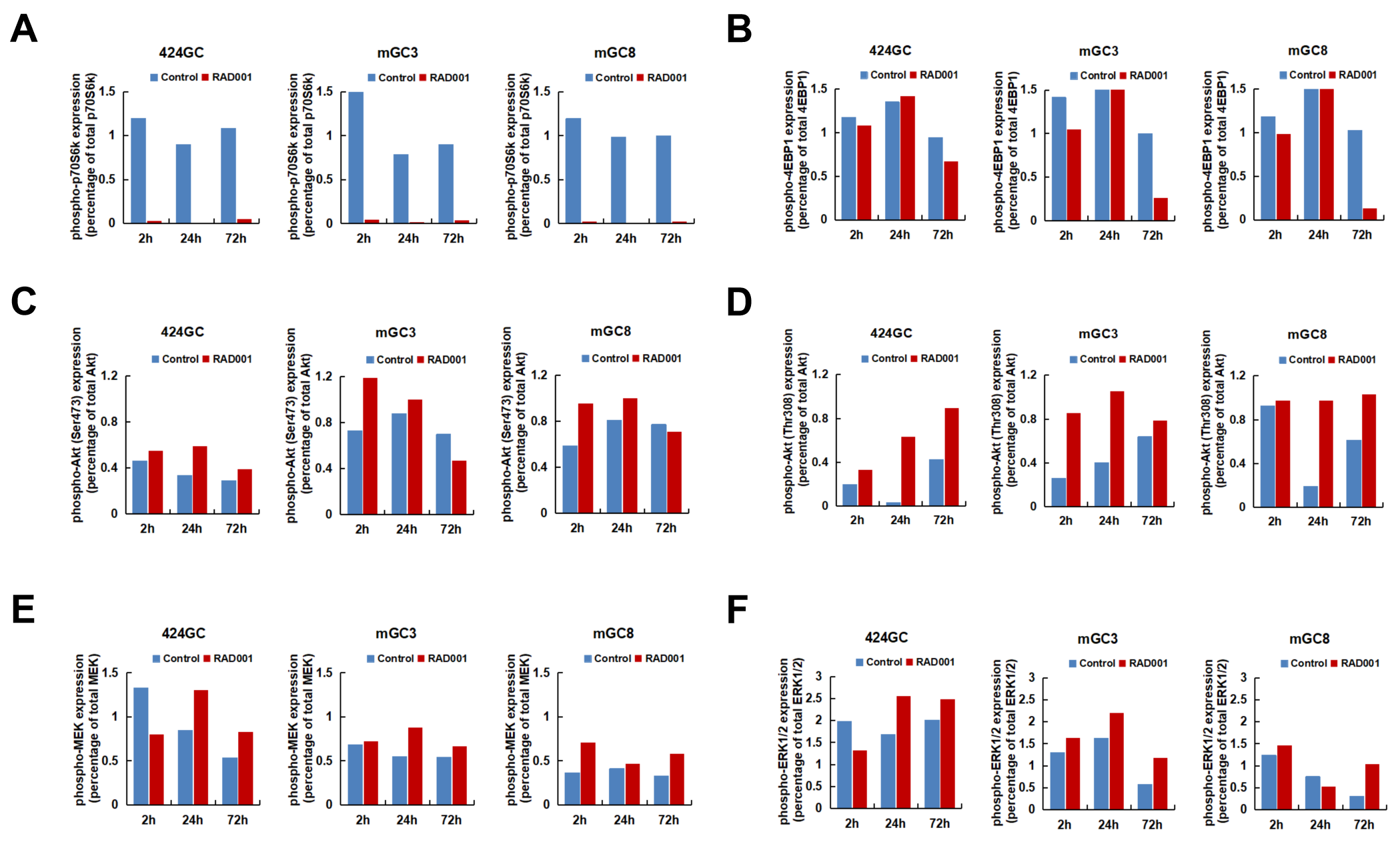

Supplement: Figure S1 — Histograms of the western blot analyses shown in Firure 4A for short term treatment of 2h, 24h and 72h in the cell line 424GC, mGC3 and mGC8. Blue: control group; red: cells treated by 100nM RAD001. [file Image_1.TIF]

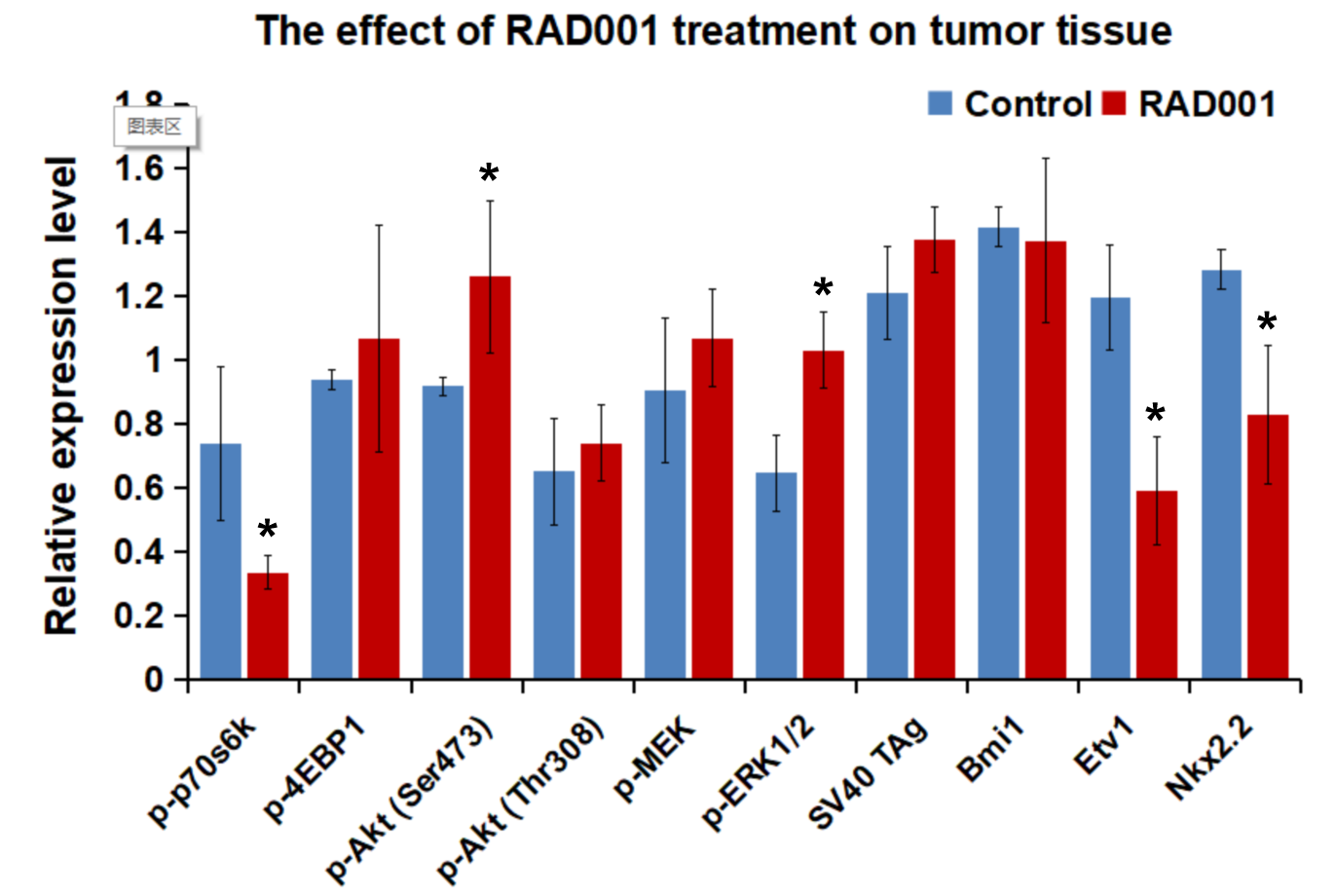

Supplement: Figure S2 — Histograms of the western blot analyses shown in Figure 4B for RAD001 treatment in vivo. The expression of phosphorylated and total p70S6K, 4EBP1, Akt, MEK and ERK1/2, as well as the expression of SV40 TAg, Bmi1, Etvi and Nkx2.2 were evaluated in the tumor tissues from RAD001 treated mice and control mice after 48 days of treatment with RAD001 or placebo (*p<0.05: RAD001 treated group versus control group). [file Image_2.TIF]

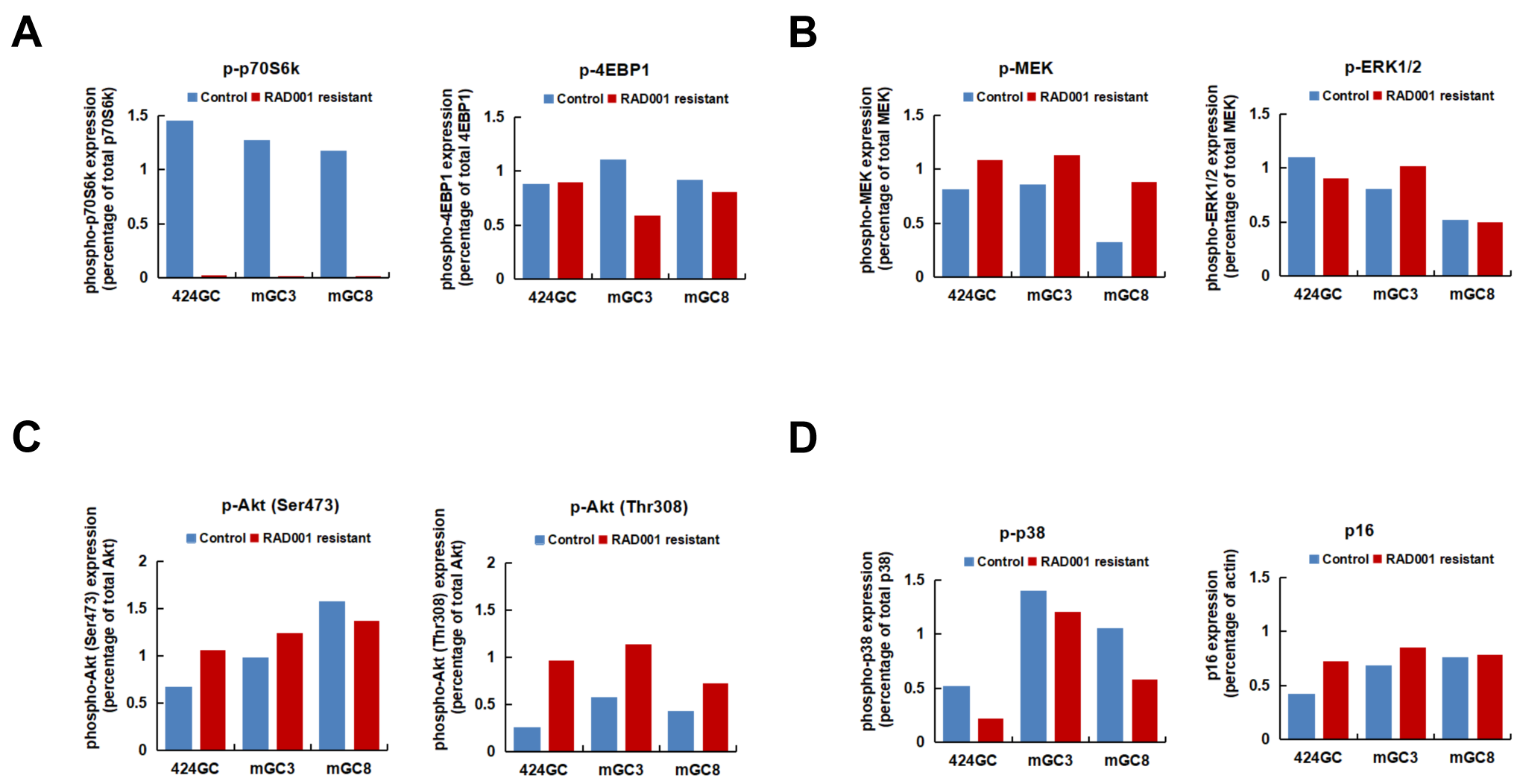

Supplement: Figure S3 — Histograms of the western blot analyses between primary cell lines and RAD001 resistant cell lines. The expression of molecular markers was compared between the RAD001 resistant cell lines kept in 400nM RAD001 and the parental untreated cell lines. [file Image_3.TIF]

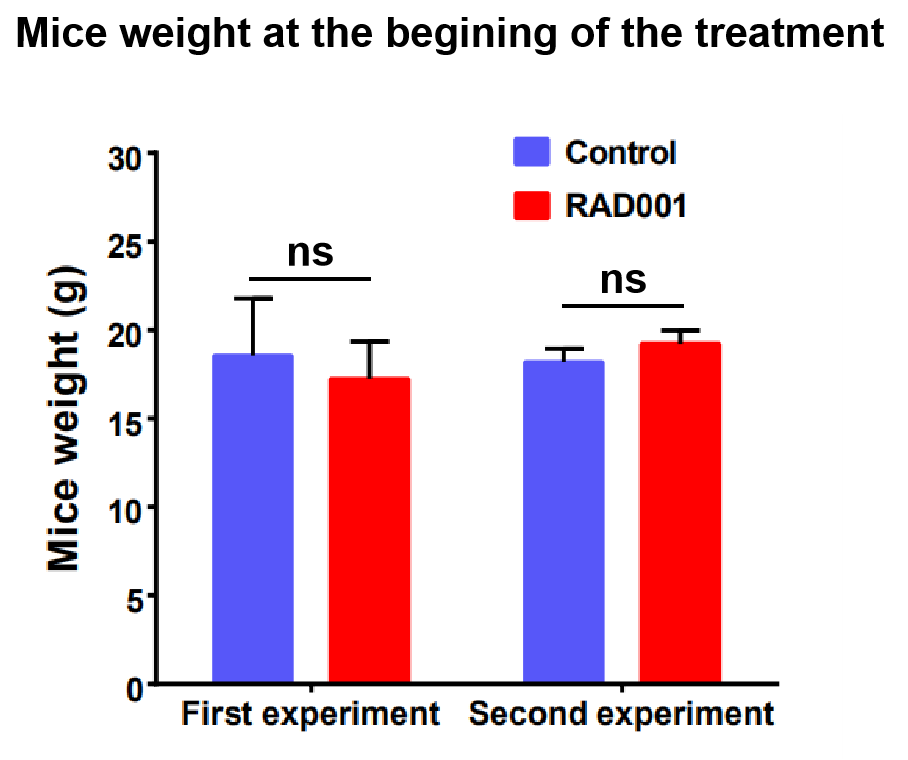

Supplement: Figure S4 — The mice weight at the beginning of the treatment. There was no significant difference of the mice weight between the control group and RAD001 treated group in both the first experiment and the second experiment. [file Image_4.TIF]
